# Deleterious Variants in *WNT10A*, *EDAR,* and *EDA* Causing Isolated and Syndromic Tooth Agenesis: A Structural Perspective from Molecular Dynamics Simulations

**DOI:** 10.3390/ijms20215282

**Published:** 2019-10-24

**Authors:** Asia Parveen, Sher Alam Khan, Muhammad Usman Mirza, Hina Bashir, Fatima Arshad, Maria Iqbal, Waseem Ahmad, Ahsan Wahab, Amal Fiaz, Sidra Naz, Fareeha Ashraf, Tayyaba Mobeen, Salman Aziz, Syed Shoaib Ahmed, Noor Muhammad, Nehal F. Hassib, Mostafa I. Mostafa, Nagwa E. Gaboon, Roquyya Gul, Saadullah Khan, Matheus Froeyen, Muhammad Shoaib, Naveed Wasif

**Affiliations:** 1 Institute of Molecular Biology and Biotechnology (IMBB), Center for Research in Molecular Medicine (CRiMM), The University of Lahore, Lahore 54000, Pakistan; asiaemaan08@gmail.com (A.P.); hina_dentist@yahoo.com (H.B.); fatimarshd968@gmail.com (F.A.); maria00415@yahoo.com (M.I.); mr.wasee@gmail.com (W.A.); amalfiaz@gmail.com (A.F.); sidra.naz557@gmail.com (S.N.); fareehaashraf274@gmail.com (F.A.); tayyabach5@gmail.com (T.M.); solodux@gmail.com (S.S.A.); 2 Faculty of Life Sciences, University of Central Punjab (UCP), Lahore 54000, Pakistan; 3 Department of Biotechnology and Genetic Engineering, Kohat University of Science and Technology (KUST), Kohat 26000, Khyber Pakhtunkhwa, Pakistan; sakmarwat79@gmail.com (S.A.K.); noormwazir@yahoo.com (N.M.); saadkhanwazir@gmail.com (S.K.); 4 Department of Pharmaceutical Sciences, REGA Institute for Medical Research, Medicinal Chemistry, University of Leuven, 3000 Leuven, Belgium; usmanmirzapk@yahoo.com (M.U.M.); mathy.froeyen@kuleuven.be (M.F.); 5 Department of Biochemistry, Sharif Medical and Dental College, Lahore 54000, Pakistan; 6 Baptist Medical Center, University of Alabama at Birmingham, Montgomery, AL 35294, USA; Drahsan.wahab@gmail.com; 7 Department of Allied Health Sciences, Superior University, Lahore 54000, Pakistan; 8 Institute of Advanced Dental Sciences and Research, Lahore 54000, Pakistan; drsalmanaziz@gmail.com; 9 Dental Section, Azra Naheed Medical College, Superior University, Lahore 54000, Pakistan; 10 Orodental Genetics Department, National Research Centre, Giza 12622, Egypt; nounih@hotmail.com (N.F.H.); mostafanrc@yahoo.com (M.I.M.); 11 Medical Genetics Center, Faculty of Medicine, AinShams University, Cairo 12413, Egypt; nogaota5000@yahoo.com; 12 Faculty of Life Sciences, Gulab Devi Educational Complex, Lahore, Ferozepur Road, Lahore 54000, Pakistan; roquyya.gul@gdec.edu.pk; 13 Biotech Research and Innovation Center (BRIC), Faculty of Health and Medical Sciences, University of Copenhagen, 2200 Copenhagen, Denmark; muhammad.shoaib@bric.ku.dk; 14 Institute of Human Genetics, University of Ulm, 89081 Ulm, Germany; 15 Institute of Human Genetics, University Hospital Schleswig-Holstein, Campus Kiel, D-24105 Kiel, Germany

**Keywords:** Hypodontia/oligodontia, Hypohidrotic ectodermal dysplasia, *WNT10A*, *EDAR*, *EDA*, exome sequencing, MD simulations

## Abstract

The dental abnormalities are the typical features of many ectodermal dysplasias along with congenital malformations of nails, skin, hair, and sweat glands. However, several reports of non-syndromic/isolated tooth agenesis have also been found in the literature. The characteristic features of hypohidrotic ectodermal dysplasia (HED) comprise of hypodontia/oligodontia, along with hypohidrosis/anhidrosis, and hypotrichosis. Pathogenic variants in *EDA*, *EDAR*, *EDARADD*, and *TRAF6*, cause the phenotypic expression of HED. Genetic alterations in *EDA* and *WNT10A* cause particularly non-syndromic/isolated oligodontia. In the current project, we recruited 57 patients of 17 genetic pedigrees (A-Q) from different geographic regions of the world, including Pakistan, Egypt, Saudi Arabia, and Syria. The molecular investigation of different syndromic and non-syndromic dental conditions, including hypodontia, oligodontia, generalized odontodysplasia, and dental crowding was carried out by using exome and Sanger sequencing. We have identified a novel missense variant (c.311G>A; p.Arg104His) in *WNT10A* in three oligodontia patients of family A, two novel sequence variants (c.207delinsTT, p.Gly70Trpfs*25 and c.1300T>G; p.Try434Gly) in *EDAR* in three patients of family B and four patients of family C, respectively. To better understand the structural and functional consequences of missense variants in WNT10A and EDAR on the stability of the proteins, we have performed extensive molecular dynamic (MD) simulations. We have also identified three previously reported pathogenic variants (c.1076T>C; p.Met359Thr), (c.1133C>T; p.Thr378Met) and (c.594_595insC; Gly201Argfs*39) in *EDA* in family D (four patients), E (two patients) and F (one patient), correspondingly. Presently, our data explain the genetic cause of 18 syndromic and non-syndromic tooth agenesis patients in six autosomal recessive and X-linked pedigrees (A-F), which expand the mutational spectrum of these unique clinical manifestations.

## 1. Introduction

The developmental abnormalities of the ectodermal appendages, such as hypodontia/oligodontia, hypotrichosis, and hypohidrosis are a heterogeneous group of syndromic and non-syndromic disorders [1]. Hypodontia and oligodontia are the clinical manifestations of many syndromic ectodermal dysplasias (EDs). However, the isolated cases are also frequently reported. Pathogenic alterations in *MSX1* (Msh homeobox-1, OMIM *142983), *PAX9* (Paired box gene-9, OMIM *167416), *AXIN2* (Axis Inhibitor-2, OMIM *604025), *EDA* (Ectodysplasin-A, OMIM *300451), *WNT10A* (Wingless-Type MMTV Integration Site Family, Member 10a, OMIM *606268), *LRP6* (Low Density Lipoprotein Receptor-Related Protein-6, OMIM *616724), *KREMEN1* (Kringle Domain-Containing Transmembrane Protein-1, OMIM *609898) have been reported in the literature to cause non-syndromic hypodontia/oligodontia [2,3,4,5,6,7].

Hypohidrotic ectodermal dysplasia (HED) is the most commonly known ED, which carries all the modes of Mendelian inheritance (autosomal recessive/dominant OMIM #224900, #129490 and X-linked OMIM #305100) [8]. HED is characterized by sparse-to-absent hair (hypotrichosis), partial or complete absence of dentition (hypodontia/oligodontia/anodontia) with the conical appearance and lack of sweating (hypohidrosis/anhydrosis), because of malfunctioning sweat glands along with other features of skin pigmentation and prominent protruded lips [9,10,11,12]. Heterozygous or biallelic variations in specific genes, including *EDA* [Ectodysplasin-A, OMIM *300451), *EDAR* (Ectodysplasin-A receptor, OMIM *604095], *EDARADD* [Ectodysplasin-A Receptor Associated Death-Domain, OMIM *606603], *IKBKG* [Inhibitor of Nuclear Factor Kappa B (*NF-kB*) Kinase Subunit Gamma, OMIM *300248] and *TRAF6* [Tumour Necrosis Factor (*TNF*) receptor-associated factor 6, OMIM *602355] have been reported to cause the pathogenicity of HED.

EDA is a soluble ligand-protein encoded by *EDA*. The C-terminal TNF homology domain of EDA interacts with the transmembrane receptor protein EDAR, which is an ectodysplasin-A anhidrotic receptor [13]. The recruitment of EDARADD protein as an adaptor with EDAR activates the *NF-kB* signaling [3,14,15,16]. Different modes of inheritance of HED are associated with the genetic variants in *EDA* (X-linked), *EDAR*, *EDARADD* (autosomal recessive and dominant) [3,17,18,19,20,21]. Mutations in *WNT10A* disrupt the Wnt-signaling pathway, causing autosomal dominant and recessive clinical conditions like Odonto-onycho-dermal dysplasia (OMIM #257980), Schopf-Schulz-Passarge syndrome (OMIM #224750) and isolated tooth agenesis (OMIM #150400) [22,23,24,25,26,27].

The present study was designed to explore the disease-causing variants in 57 patients of 17 genetic pedigrees (A-Q) recruited from various parts of the world and to understand the impact of these genetic alterations on the protein stability. The patients showed distinct dental conditions with, and without, associated phenotypic manifestations. Exome and Sanger sequencing data revealed three novel and three already reported deleterious variants in 18 patients of two oligodontia (A, D) and four HED (B, C, E, F) families. 

Over the years, molecular dynamics (MD) simulations pondered to be a reliable method in exploring the dynamic consequences and underlying structural effects due to mutations [28,29,30,31,32]. In the current study, the structural consequences of the novel missense variants on the respective protein stability were carried out using long-run MD simulations followed by interaction energetics with the binding proteins, which allowed us to study the protein conformational characteristics at every step during MD simulations [33,34]. The findings of our MD analysis provide new insights into the structural basis of novel disease-causing variants and offer a possible molecular explanation on the protein function of *WNT10A* and *EDAR* due to the novel nonsynonymous variations.

## 2. Results

### 2.1. Clinical Findings

**Figure 1:**
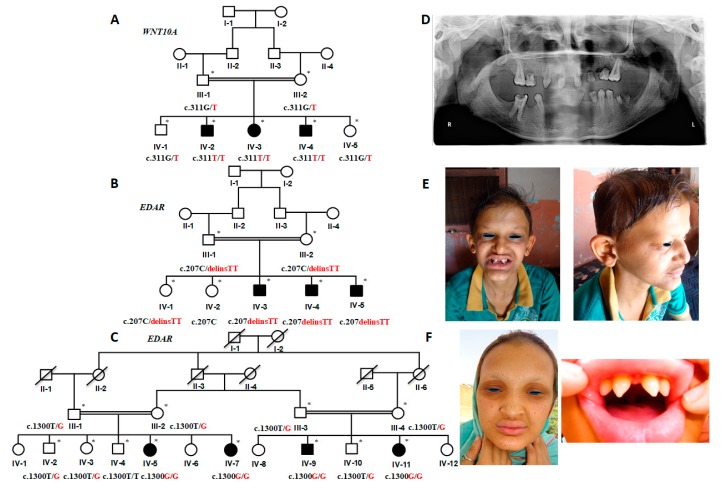
(**A**–**C**) Pedigrees of family A, B, and C show the autosomal recessive mode of inheritance. The available samples for the genetic analyses are marked with asterisks (*). The genotypes of each participant in these pedigrees are mentioned below the symbols, showing the segregation of the alleles. The black color represents the wild-type allele and red color depicts the disease-allele (**D**) Orthopantomogram (OPG) of the affected member (IV-2), family A, revealed the absence of the majority of permanent teeth, except maxillary first molars, maxillary central incisors, maxillary right canine, and mandibular left first and second premolars and mandibular right first premolar (teeth # 16, 26, 11, 21, 13, 34, 35 and 44, respectively, according to FDI nomenclature). (**E**,**F**) A male patient (IV-3) of family B and a female patient (IV-5) of family C are showing pigmentation around lips and eyes, sparse scalp hair, eye-brows, and eye-lashes, saddle-shaped nose, and characteristic canonical teeth.

Isolated oligodontia phenotype was observed in patients (IV-2, IV-3, IV-4) of consanguineous family A (Figure 1A), exhibiting an autosomal recessive mode of inheritance. Orthopantomogram (OPG) of a 25 years old affected member (IV-2) shows the absence of all permanent teeth except maxillary first molars (teeth # 16 & 26 FDI), malformed/hypoplastic maxillary central incisors (teeth # 11 & 21 FDI) and maxillary right canine (# 13 FDI). In the mandibular arch, both left permanent first and second premolars (# 34 & 35 FDI) are present, while only the right first premolar (# 44 FDI) is evident. The mandibular right first molar (# 46 FDI) is the last standing tooth in the mandibular right quadrant. The decayed root stumps of mandibular left first molar are visible, which is depictive of the fact that although this tooth developed initially but was destroyed by dental caries. The teeth # 55, 63, 65, and # 74 (all FDI) were the retaining deciduous teeth. The tooth buds of most of the permanent teeth are absent, suggesting that they are congenitally missing (Figure 1D).

Initially, patient IV-2 complained of inadequate sweating. However, the other affected members IV-3, IV4 did not record any such deficiency during their interviews. The dermatologists examined all the patients at the University Hospital, the University of Lahore, and the clinical conditions like palmoplantar keratoderma, hypo/hyperhidrosis, hypotrichosis, and nail dysplasias were thoroughly excluded.

The clinical features of the patients (IV-3, IV-4, IV-5) of family B (Figure 1B) and the patients (IV-5, IV-7, IV-9, IV-11) of family C (Figure 1C), included oligodontia, sparse scalp hair, sparse-to-absent eye-brows and eye-lashes, depressed nasal bridge resulting in saddle-shaped nose, frontal bossing, everted and prominent lips, wrinkles, and hyperpigmentation around the eyes (Figure 1E,F). In family C, the affected female members (IV-5, IV-7, IV-11) showed underdeveloped breasts and frequent irritation of eye and skin, while the mild hyper-keratoderma was observed in all the affected members (IV-5, IV-7, IV-9, IV-11). The patients of both families had complaints about the complete absence of sweating and hyperthermia, especially during the summer season.

Affected members (IV-3, IV-4, IV-6, V-2, V-3) of family D (Figure S1A) showed oligodontia phenotype. The OPG analysis of patient IV-4 explained the absence of, both maxillary and mandibular lateral incisors (teeth # 12, 22 & 32, 42 FDI), mandibular central incisors (teeth # 31& 41) and second premolars (teeth # 35 & 45). The teeth mentioned above were neither impacted, nor was there any tooth bud present. The retained deciduous teeth were visible in the OPG (teeth # 65, 71, 73, 75, 81 & 85) (Figure S1B). The defects of other ectodermal appendages were not detected during the clinical investigation.

Pedigrees E and F presented an X-linked mode of inheritance. Affected individuals (II-3, II-4) (Figure S2A) and (II-2) (Figure S3A) of both families, exhibited clinical phenotypes of HED (Figure S2B, Figure S3B), as described in affected members of family B and C.

### 2.2. Variant Screening and Pathogenicity

**Figure 2:**
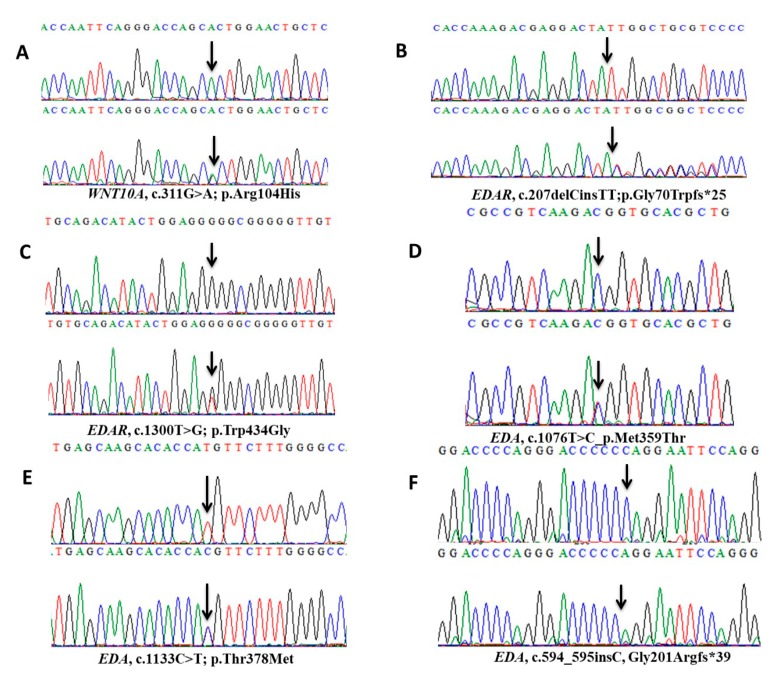
Sequence analysis of *WNT10A*, *EDAR*, and *EDA* variants. (**A**) Panel A shows a partial sequence ofexon-4 of *WNT10A* in an affected member (IV-2) with novel homozygous missense variant (c.311G>A, p.Arg104His) and of an unaffected heterozygous carrier (III-2) in family A. (**B**) Panel B presents a partial sequence of exon-4 of *EDAR* in an affected (IV-3) and an unaffected member (III-1) of family B showing a novel frameshift variant (c.207delinsTT, p.Gly70Trpfs*25), where the homozygous deletion of C nucleotide and the simultaneous insertion of TT nucleotides is evident in the affected member sequence. (**C**) Panel C is showing a partial DNA sequence of exon-12 of *EDAR* representing a novel missense variant (c.1300T>G, p.Trp434Gly) in an affected member (IV-5) and a heterozygous carrier (III-1) of family C (**D**–**F**) Three *EDA* sequence variants are presented in panel D, E and F. Panel D and E are showing missense sequence variants (c.1076T>A, p.Met359Thr; c.1133C>T, p.Thr378Met) in affected members (IV-4) (II-3) and unaffected carriers (III-2) (I-2) of family D and E, respectively. The panel F is showing a de novo frameshift variant (c.594_595insC, Gly201Argfs*39) in the affected member (II-2) of family Fwhile themother (I-2) of this affected member shows a wild-type sequence in the same panel.

Exome sequencing analysis revealed a novel *WNT10A* G to A transition at nucleotide position 311 (c.311G>A) in exon-2 in an affected member (IV-2) of family A. Sanger sequencing of this variant (c.311G>A; p.Arg104His) (Figure 2A) in other affected (IV-3, IV-4) and unaffected (III-1, III-2, IV-1, IV-5) members of the family proved its co-segregation with the disease phenotype. This variant lies in a 7.9MB region of homozygosity (ROH) and is reported as a rare event by gnomAD (gnomAD, http://gnomad-old.broadinstitute.org/), with an allele frequency of 2.437e-5 (where the number of homozygote alleles is 0, the number of heterozygote alleles is 6, and the total allele number is 246,170). Heterozygote allele count for this variant was 3 out of 30,780 alleles in South Asian and 3 out of 11,626 alleles in European (Non-Finnish) populations. A ClinVar accession ID (VCV000633837.1; https://www.ncbi.nlm.nih.gov/clinvar/variation/633837/) has been assigned to this rare variant.

Mutation analysis of coding regions of *EDAR* in affected and unaffected members of family B and C detected a novel frameshift variant (c.207delinsTT, p.Gly70Trpfs*25) in exon-4 (Figure 2B) and a novel missense variant (c.1300T>G; p.Trp434Gly) in exon-12 (Figure 2C), respectively. There were no reports of the minor allele frequency of both variants in gnomAD. The frameshift variant in exon-4 of *EDAR*, where deletion of nucleotide C and simultaneous insertion of nucleotides TT took place at position 207, eventually shifting the frame from glycine to tryptophan at amino acid position 70, thus having a deleterious effect on the structure and likely on the function of the protein. To observe the frameshift in the heterozygous carrier sequence (Figure S4A,B), we have designed the wild-type and mutant DNA strands from the point of mutation (Figure S4C). We have also generated wild-type and mutant cDNA and protein sequences (Figure S4D,E) by using the online algorithm Mutationtaster (http://mutationtaster.org/), which also indicates the production of a truncated EDAR protein because of a premature termination codon (Figure S4E). ClinVar accession numbers (VCV000633838.1; https://www.ncbi.nlm.nih.gov/clinvar/variation/633838/) (VCV000633839.1; https://www.ncbi.nlm.nih.gov/clinvar/variation/633839/) for these rare *EDAR* variants have been approved recently.

The online prediction algorithms like MutationTaster, PROVEAN, SIFT, PolyPhen 2.0, and I-Mutant 3.0 have classified the novel missense variants in *WNT10A* and *EDAR* as disease-causing and predicted to decrease the protein stability. The wild-type amino acids in WNT10A (p.Arg104) and EDAR (p.Trp434) are highly conserved across the species (Figure S5A,B).

The DNA sequencing of available affected and unaffected members of family D and E identified two already known missense variants (c.1076T>C; p.Met359Thr) (Figure 2D) and (c.1133C>T; p.Thr378Met) (Figure 2E) in *EDA*. A previously described de novo frameshift change (c.594_595insC, Gly201Argfs*39) (Figure 2F) was identified in the affected member of family F. The involvement of all other known genes including *MSX1*, *PAX9*, *AXIN2*, *EDARADD*, *LRP6*, *KREMEN1*, *TRAF6* and *NFκB* (*NEMO*) was excluded by direct Sanger sequencing and/or from the exome sequencing data in all 57 patients of diverse tooth anomalies. 

### 2.3. Protein Structure and Stability Prediction

The SWISS-MODEL was used to generate protein structure models with high accuracy. Xenopus WNT8 (PDB ID: 4F0A.B; Sequence identity: 40.3%) was used as a template to model human-WNT10A whereas, EDAR and EDARADD were modeled using TNF receptor superfamily member 16 (PDB ID: 2N97.A; Sequence identity: 32.93%), and Interleukin-1 receptor-associated kinase 4 (PDB ID: 3MOP.G; Sequence identity: 28.72%) as templates, respectively. The Ramachandran evaluations estimated the reliability of structures by using Molprobity and ProSA web server, after 20ns MD-assisted model refinement (Table 1). Later, the protein structure stability was estimated from various structure-based servers, which predicted the variants as destabilizing, based on ΔΔG predictions (Table 2). 

**Figure 3:**
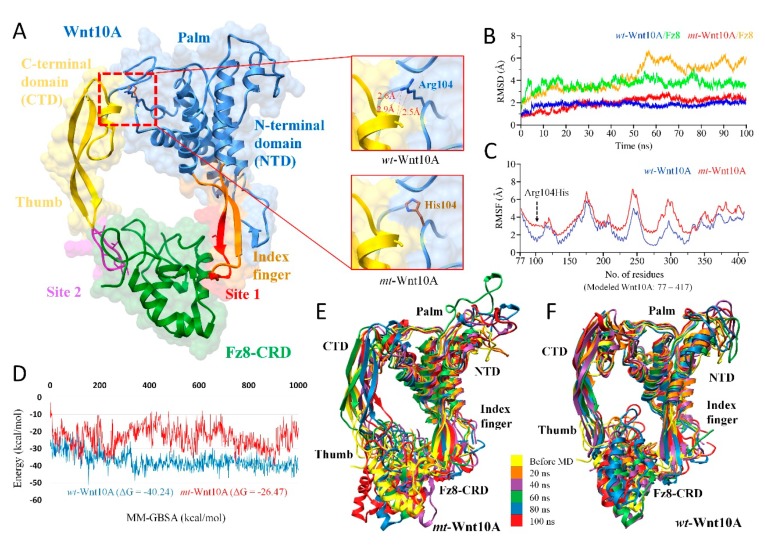
Structural analysis of WNT10A complexed with Fz8-CRD. (**A**) Overall structural representation of WNT10A in complex with Fz8-CRD followed by a close-up of interatomic interactions with a substitution of Arg104His in WNT10A. Ribbon representations of domains with distinct color as N-terminal index finger (brown), central palm (cauliflower) and C-terminal thumb (gold) domains, while *Fz8-CRD* is displayed in green. (**B**) RMSD trajectories of WNT10A Cα-backbone atoms of mutant (red) and wild-type (blue) complexed with *Fz8-CRD* throughout 100ns. (**C**) RMSF trajectories of mutant (red) and wild-type (blue). (**D**) MM-GBSA trajectories (total of 1000 snapshots from the 100ns MD simulations) of mutant (red) and wild-type (blue) (**E**,**F**). Structural conformations of WNT10A/Fz8-CRD obtained after 20ns, 40ns, 60ns, 80ns, and 100ns are superimposed together.

**Figure 4:**
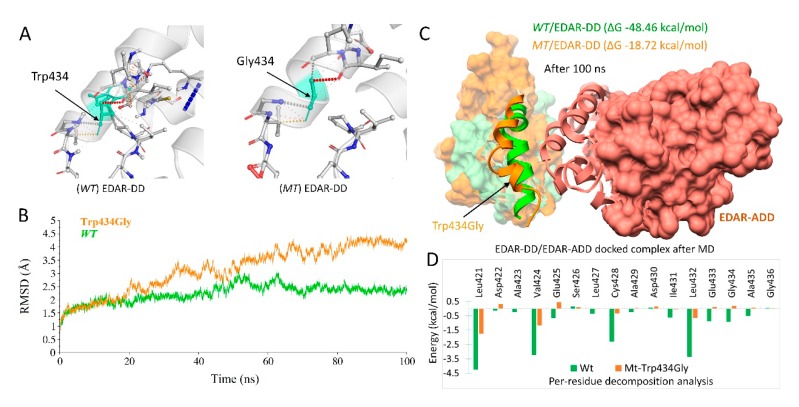
Structural analysis of EDAR complexed with EDARADD. (**A**) Interatomic interactions with a substitution of Trp to Gly at position 434 in EDAR. (**B**) RMSD trajectories of Cα-backbone atoms of mutant (brown) and wild-type (green) throughout 100ns. (**C**) Docked conformation of EDAR/EDARADD complex after 100 ns MD simulation, with exposed ribbon conformation of 5^th^ α-helix of wt (green) and mt-EDAR (brown). (**D**) Per-residue decomposition analysis of 5^th^ α-helix with the same color code as above.

ClusPro generated near-native conformations of the WNT10A/Fz8-CRD, and EDAR/EDARADD docked complexes which revealed highly favorable results based on the lowest energy values of balanced, electrostatic, hydrophobic and VdW+Elec (Table S1). In case of WNT10A/Fz8-CRD, the near-native conformation was evident after the structural alignment with the *XWNT8/Murine-Fz8-CRD* and predicted to have the same distinctive donut shape [35], where *Fz8-CRD* was clenched by two opposing sites including the N-terminal index finger (Site 1) and C-terminal thumb (Site 2) projected from the central palm domain as shown in Figure 3. Whereas, there was no reported information about the structural conformation of the EDAR/EDARADD complex, therefore, a representative structure from the largest cluster in all four categories (Table S1) was retrieved and compared, and the most consistent conformation was selected for further analysis. All the molecular modeling analysis of EDAR/EDARADD is displayed in Figure 4.

The quantitative stability changes (ΔΔG) were predicted using multiple programs to see the impact of individual missense variants on the protein function and stability. DUET was utilized to calculate the protein structure stability changes upon variations, which predicted Arg104His in WNT10A and Trp434Gly in EDAR as destabilizing with negative free energy change (ΔΔG) values −1.431 kcal/mol and −2.762 kcal/mol, respectively. Furthermore, the estimated vibrational entropy energy change (ΔΔS_Vib_) and thermal stability (ΔΔG) as calculated from ENCoM server [36], also indicated the increase of flexibility in WNT10A (ΔΔS_Vib_ ENCoM: 0.523 kcal.mol^−1^.K^−1^; ΔΔG: −0.348 kcal/mol as destabilizing) and, EDAR (ΔΔS_Vib_ ENCoM: 1.661 kcal.mol^−1^.K^−1^; ΔΔG: −1.329 kcal/mol as destabilizing).

### 2.4. MD Simulations and Interpretations of Novel Missense Variants

To prove the hypothesis of the increased flexibility upon alteration at an atomistic level, we carried out 100ns MD simulations on the mutated WNT10-A/Fz8-CRD and EDAR/EDARADD docked complexes together with wild-type conformations. The structural stability during simulations was observed by computing the RMSD values of all backbone atoms for the mutated and wild-type structures.

#### 2.4.1. WNT10A Missense Variant (c.311G>A; p.Arg104His)

The reliability of the binding conformation was evident from the crystal structure of XWnt8 complexed with the Fz8-CRD, which is reported as a model system to study Wnt/Fz interactions because it binds to and activates mammalian Fz [37]. The variant p.Arg104His was present in the central palm domain, which configures the projected N-terminal index finger and C-terminal thumb domain to interact with Fz8-CRD (Figure 3A). The Arg104 was found crucial in establishing three H-bonds (atomic distance <3 Å) with Cys and Gly of the thumb domain and buttressed the configuration of the thumb domain (Figure 3A). However, the replacement to His104 abolished these important interactions and resulted in the increased flexibility of the thumb domain, which was also evident from the vibrational entropy energy (ΔΔS_Vib_ ENCoM: 0.523 kcal.mol^−1^.K^−1^. 

The RMSD trajectories of the WNT10-A/Fz8-CRD complex throughout 100ns are displayed in Figure 3B. The wt-WNT10-A remained stable throughout the simulation; however, the smallest fluctuations were seen (< 0.5 Å), which affect the Fz8-CRD stability up to ~1.5 Å. In comparison, the RMSD of mt-WNT10-A fluctuated around 1.5 Å from an initial structure which triggered higher fluctuations inbound *Fz8-CRD* till 60ns to a value around ~5 Å. The more stable RMSD of wt-WNT10-A/Fz8-CRD indicated that the compact conformation of the complex was primarily preserved due to Arg104, which showed a considerable difference with a substitution to His104. To better explore this difference, root mean square fluctuations (RMSFs) were analyzed throughout 100ns, which highlighted the flexible regions of *wt* and *mt*-WNT10-A (Figure 3C). The most pronounced Cα-RMSF differences occurred for residues 90 to 110 and in the C-terminal thumb domain. The replacement of Arg104 to His104 elicited substantial mobility, which retained its impact on the other regions, significantly on the C-terminal thumb domain (Figure 3C).

Thereafter, the MM-GBSA binding free energy calculations were performed (total of 1000 snapshots from the 100 ns) to explore the overall binding energy difference of the complex upon variation. The overall MM-GBSA of mt-WNT10-A/Fz8-CRD showed unstable ΔG_tol_ (free energies of binding in kcal/mol) trajectory as compared to wt-WNT10-A/Fz8-CRD, and the considerable difference was seen in the *mt* (Avg ΔG_tol_ = −26.47 kcal/mol) and *wt* (ΔG_tol_ = −40.24 kcal/mol) complexes (Figure 3D). This variation in the binding free energy was evident from the destabilizing thumb domain for 20, 40, 60, 80, and 100ns, as shown in Figure 3E,F. The extent of the conformational shift of thumb domain in the mt-WNT10-A complexed with the Fz8-*CRD* can also be seen as compared to its wild-type complex, which remained stable and converged throughout the simulation as shown in Figure 3E,F. 

#### 2.4.2. EDAR Missense Variant (c.1300T>G; p.Trp434Gly)

The interatomic interactions in the starting structure of EDAR with a substitution of Trp to Gly revealed a significant difference in several interactions, which may lead to disruption in helicity, as shown in Figure 4A. Throughout MD simulations, wt and mt-EDAR complexed with EDARADD remained stable during the starting 20ns and showed similar patterns of deviation and convergence between 1 to 2 Å RMSD value. Later, the mt-EDAR showed a gradual increase in RMSD throughout simulations from 20 to 100ns and exhibited deviation of up to ~4.5 Å, reflecting a more destabilizing effect while wt-EDAR remained stable for last ~25ns (converged between 2 to 2.5 Å) (Figure 4B). 

To explore the mutational effect on mt-EDAR/EDARADD complex, we extracted a PDB structure after 100ns. Figure 4C displayed the structural effect upon alteration, where the conformation of the last 5^th^ α-helix (Leu421-Gly436) at C-terminal differs significantly as compared to wild-type. This distortion was induced due to the replacement of a highly interacted aromatic Trp to Gly, which may lead to a decrease in the overall stability of the 5^th^ α-helix Figure 4A. To gain better insight into the interaction energies of EDAR (wt/mt) with EDARADD, MM-GBSA binding free energies were calculated using the MM-GBSA module of Amber 16. Overall, as shown in Figure 4C, the MM-GBSA ΔG_tol_ of the wt and mt-EDAR were found to be −48.46 and −18.72 kcal/mol, respectively. The wt-EDAR revealed the highest ability to bind to EDARADD as compared to mt-EDAR, which instigated the binding ability to decrease significantly. This substantial fluctuation in the binding energy of EDAR (wt/mt) was further investigated by per-residue decomposition analysis of 5^th^ α-helix residues from Leu421–Gly436, as shown in Figure 4D. As illustrated, it was obvious that all residues of the 5^th^ α-helix of wt-EDAR showed favorable binding free energies. The ΔG_tol_ of residues, Leu421 (−4.25 kcal/mol), Val424 (−3.23 kcal/mol), Cys428 (−2.28 kcal/mol) and Leu432 (−3.35 kcal/mol) which decreased to −1.74, −1.17, −0.34 and −0.645 kcal/mol in mt-EDAR, respectively. Notably, the mutated residue Gly showed absolute unfavorable binding free energies (ΔG_tol_ = +0.18 kcal/mol) along with other neighboring residues including, Glu433 (ΔG_tol_ = +0.11 kcal/mol), Glu425 (ΔG_tol_ = +0.45 kcal/mol) and Asp422 (ΔG_tol_ = +0.31 kcal/mol) as compared to wt-EDAR.

## 3. Discussion

The exome and Sanger sequencing analyses revealed pathogenic variants in *WNT10A*, *EDAR*, and *EDA* in 18 patients of six autosomal recessive and X-linked pedigrees (A-F). These patients presented oligodontia/hypodontia (Pedigree A, D) and HED (Pedigree B, C, E, F). 

Exome sequencing combined with DNA sequencing of affected and unaffected members of family A revealed a novel missense variant (c.311G>A; p.Arg104His) in *WNT10A*. *WNT10A* is involved in the Wnt/β-catenin pathway, which regulates the skin and teeth embryogenesis, hair follicle morphogenesis, and teeth renewal [38,39,40]. *WNT10A* pathogenic variants have been reported to cause highly variable disease manifestations, including dry hair, pilar keratosis, palmar erythema and keratoderma, severe hypodontia, onychodysplasia, smooth tongue, and hyperhidrosis of palms and soles, sparse eye-brows, dystrophic finger and toenails [22]. *WNT10A* has an active involvement in the embryogenesis of teeth, and it establishes a strong interaction between the dental epithelium and the underlying mesenchyme [41]. According to the Human Gene Mutation Database (HGMD Professional, 2018.3), eighty-four *WNT10A* genetic alterations have been reported in syndromic and non-syndromic tooth agenesis phenotypes and EDs. Only thirty-eight variants are enlisted in *WNT10A* for isolated tooth agenesis phenotypes. The novel variant (c.311G>A; p.Arg104His), identified in the current study, is also causing autosomal recessive isolated oligodontia in a Pakistani family. 

Pathogenic alterations of *EDAR* are responsible for causing typical manifestations of HED. We have identified two novel variants in autosomal recessive families B and C, belonging to Punjab and Khyber Pakhtunkhwa provinces of Pakistan. A frameshift variant (c.207delinsTT, p.Gly70Trpfs*25) in family B, while a missense variant (c.1300T>G; p.Trp434Gly) in family C, co-segregated with the disease phenotype. HGMD Professional, 2018.3 has described sixty-two pathogenic variants in *EDAR*. Fifty of these reported variants testify the HED phenotype in patients around the world. The identified novel frameshift variant (c.207delinsTT, p.Gly70Trpfs*25) in *EDAR* introduced a premature termination codon that is expected to cause a non-sense mediated decay (NMD), leading to the loss of function of EDAR protein. Hentze and Kulozik, 1999 have described that the imperfect messages initiated by truncating mutations are eliminated by NMD [42].The TGG codon (p.Trp434) of EDAR appears to be highly variable; the two disease-causing genetic alterations have already been reported in it. Chassaing et al. 2006 have identified the first variant TGG^Trp^-TGT^Cys^, (c.1302G>T, p.Trp434Cys) in a patient of French origin and Shimomura et al. 2009 have reported the second variant TGG^Trp^-CGG^Arg^ (c.1300T>C, p.Trp434Arg) in a Pakistani family [43,44]. Here, we report a third variant in the same codon TGG^Trp^-GGG^Gly^ (c.1300T>G; p.Trp434Gly) causing HED phenotype in another autosomal recessive Pakistani family. 

In the current investigations, the impact of two novel missense variants in WNT10A and EDAR on the stability of the proteins was also estimated through a series of computational methods to improve the predictions. The protein structure stability and the dynamic consequences upon variations were evaluated using MD simulation. The MD simulation generated a stable system, that can be assessed from the RMSD values of the wild-type structures of WNT10A and EDAR, which fluctuated around 3 Å and 6 Å from the starting structure and the system attained equilibrium and the fluctuation reduced to ~1.5 Å, and 3 Å, respectively. This stable system was suitable for monitoring the dynamic stability of their corresponding mutated structures. 

The gradual decrease in binding free energy was expected due to the change from Arg to His and Trp to Gly, which replaced a charged amino acid to a polar one in WNT10A, and a large and highly interacting side chain to a smaller hydrophobic residue in EDAR. These substitutions might eliminate significant electrostatic interactions with the adjacent residues as well. 

The WNT10A variant p.Arg104His was expected to abolish the essential hydrogen bonds with the Cys and Gly residues of the thumb domain (Figure 3A), which eventually led to a significant deviation from the starting structure and gradual flexibility of C-terminal thumb domain over the period of 100ns (Figure 3B,C). The fact that flexibility induces large fluctuations in bound *Fz8-CRD* (Figure 3B) may explain the finding that a small conformational change upon complex formation triggers a substantial structural change in the binding protein [45]. The overall structural flexibility in mt-WNT10A/Fz8-CRD complex was also plausible from an inconsistent binding free energy trajectory (Average ΔG_tol_ = −26.47 kcal/mol) as compared to stabilized energetics in its *wt* (Average ΔG_tol_ = −40.24 kcal/mol) (Figure 3D). This underlying phenomenon might significantly destabilize the overall binary interaction of bound *Fz8-CRD* in the *mt*-complex (Figure 3E), which is considered to be necessary for the activation of *Wnt/β-catenin* pathway and plays a crucial role at multiple stages of tooth development [41]. Additionally, this dynamic effect upon variation was evident from increased vibrational entropy energy between wild-type and mt-WNT10A (ΔΔS_Vib_: 0.523 kcal.mol^−1^.K^−1^), which escalated the flexibility of thumb domain [calculated by ENCoM [36]]. This vibrational entropy contributes significantly to the binding free energies of proteins [36,46]. 

In *wt-EDAR*, the stability of 5^th^ α-helix was achieved by the hydrogen bonds (Figure 4A), which corresponded to the high electrostatic interaction energy and exhibited favorable binding free energy (ΔG_tol_ = −48.46 kcal/mol) (Figure 4B,D). Additionally, in comparison to *wt-EDAR*, changes in the overall dynamics were observed in mt-EDAR (Figure 4C). The possible effects due to Trp434Gly substitution includes, (i) the significant decrease in the side chain volume, which might eliminate the electrostatic interactions with the adjacent side chains and reduction in number of H-bonds [47] (Figure 4A), (ii) changes in the side-chain packing and (iii) changes in water accessibility which has a vital role in helix stabilization [48,49], thus destabilizing the overall helical conformation. The increased RSMD deviation due to the decreased helicity in 5^th^ α-helix of mt-EDAR was in agreement with the destabilizing effect and vibrational entropy change as predicted from DEUT (ΔΔG = −2.762 kcal/mol) and ENCoM (ΔΔS_Vib_: 1.661 kcal.mol^−1^.K^−1^). That indicated the influence of p.Trp434Gly variant on the flexibility of the molecule and may trigger a much more significant structural change in its binding partner [50]. The binding of EDA to EDAR allows the recruitment of its adaptor protein, EDARADD, which is crucial in activating the NF-κB signaling pathway [15,51,52] and contributes to the ectodermal appendages morphogenesis [3]. The DD of EDAR has been reported to be essential for its interaction with EDARADD in building an intracellular signal transduction complex and its truncation terminates its affinity to EDARADD [53,54]. We speculated that the p.Trp434Gly substitution of *EDAR* might be a loss-of-function alteration that would significantly alter its affinity to EDARADD, and finally, influence the downstream activation of NF-κB.

The identified novel missense variants in *WNT10A* (c.311G>A; p.Arg104His) and *EDAR* (c.1300T>G; p.Trp434Gly) are assumed to cause autosomal recessive oligodontia and autosomal recessive HED. The structural analyses of WNT10A and EDAR proteins indicated that these pathogenic variants might alter the overall protein function. Therefore, we speculate that the disrupted interaction of mutated proteins may cause failure to tooth morphogenesis leading to isolated and syndromic tooth agenesis (HED). 

The *EDA* variants, two missense (c.1076T>C; p.Met359Thr, c.1133C>T p.Thr378Met) in families D and E, and a de novo frameshift (c.594_595insC, Gly201Argfs*39) in family F, have already been described in the literature [51,55,56,57,58,59,60]. Rasool et al. 2008 have hypothesized that the *EDA* variant (c.1076T>C; p.Met359Thr) may somehow affect the stability of EDA protein, as shown by molecular modeling studies [55]. The other missense variant (c.1133C>T p.Thr378Met) has been mapped on the TNF-like domain of EDA and is predicted to change its functional nature [51]. The known de novo frameshift variant (c.594_595insC, Gly201Argfs*39) is anticipated to partially destroy the collagen-like domain leading to non-functional EDA [58,61].

## 4. Material and Methods

### 4.1. Project Approval, Recruitment of Patients and DNA Extraction

This study was designed according to the principles of the Declaration of Helsinki. Permission for this research project and the enrollment of all 57 patients and healthy members was obtained from the Institutional Review Boards (IRB) and Ethics Committees of The University of Lahore (VC-UOL/240712/A01-UOL; 23.07.2012), Lahore, Pakistan, National Research Centre (10.10.2015)Giza, Egypt, King Abdul-Aziz University (Ref.No. 24/14, 13.02.2014), Jeddah Saudi Arabia and Kohat University of Science and Technology (KUST) (Ref.No.VC-KUST/ethicalcommittee/16-25, 26.04.2016), Kohat, Pakistan. Pedigree drawings and extraction of venous blood samples were performed after taking informed written consent from all the patients and available unaffected members of all 17 pedigrees (A-Q) followed by DNA extraction. 

### 4.2. Exome Sequencing

A recommended concentration of DNA (50–100 ng/ul) of one or two affected members after studying the pedigree diagrams was submitted to exome sequencing. Exome sequencing protocol and filtration criteria for the identification of rare pathogenic variants were followed as previously described [62]. The gnomAD (Genome aggregation database; http://gnomad.broadinstitute.org) was consulted at a minor allele frequency (MAF) of 0.1% as an established criterion for the rareness of variants. Rare variants were also verified in an in-house database of 511 exomes from individuals with different diseases.

### 4.3. Sanger Sequencing

After filtering the likely pathogenic variants in exome data, Sanger sequencing was performed to validate these variants. The genomic sequences, 700 bp up and downstream, of each selected variant, were obtained from the University of California Santa Cruz (UCSC) genome database browser (http://genome.ucsc.edu/cgi-bin/hgGateway), California, USA [63]. A pair of primers (Table S2) for each identified variant was used for PCR amplification. 

Direct Sanger sequencing of *EDA* and *EDAR* was also performed in specific samples. The primers were designed using AmplifX v1.5.4 software (https://inp.univ-amu.fr/en/amplifx-manage-test-and-design-your-primers-for-pcr), Marseille Cedex, France. Exo-SAP protocol, Thermo Fisher Scientific, MA, USA, was used for the cleanup of PCR products. ABI3730 genetic analyzer was used for Sanger sequencing with BigDye chemistry v3.1, Thermo Fisher Scientific, MA, USA. Sanger Sequencing data were analyzed with the SeqMan Pro (DNASTAR, Inc., Madison, WI, UK). Evolutionary conservation of the novel single amino acid variations of WNT10A (p.Arg104His) and EDAR (p.Trp434Gly) was examined in WNT10A and EDAR orthologs using http://www.ncbi.nlm.nih.gov/homologene/, Bethesda, MD, USA. 

### 4.4. Pathogenicity Context of the Missense Variants

The pathogenicity of the novel missense variants in *WNT10A* and *EDAR* was predicted through MutationTaster [64] PROVEAN [65], SIFT [66], PolyPhen 2.0 [67] and I-Mutant 3.0 [68].

### 4.5. Molecular Modeling and Protein Stability Predictions

The corresponding protein structures, including human-WNT10A and EDAR, were not present in the Protein Data Bank (PDB) [69]. Therefore, the homology modeling of wild-type and mutants was performed through SWISS-MODEL using the best template [70]. The structural assessment was performed using Molprobity [71] and ProSA [72], and the best models were considered for further structural elucidation. To better comprehend the predicted effect of the novel variants, the binding partners of WNT10A and EDAR were also considered, and protein-protein docking was employed using ClusPro (http://nrc.bu.edu/cluster), an automated protein-protein docking server. It generates RMSD-based clustering of 1000 docked conformations to find the most massive cluster that likely represents the best possible models of the complex [73]. WNT10A acts as a ligand and functions through the canonical *Wnt/β-catenin* pathway [35,41], which interacts with the N-terminal cysteine-rich domain (*CRD*) of seven-pass transmembrane receptor Frizzled (*Fz*) (STRING ID: 10090) and Low-density-lipoprotein receptor-related protein (LRP) [74]. For the current study, the *Fz* receptor was used for the interaction study, and the human *Fz8-CRD* crystal structure (PDB ID: 5CM4) was retrieved from the PDB [75]. While EDAR functions through the EDA/EDAR/NF-κB signaling pathway [76]. EDAR has a *CRD* in its extracellular region (residues from 27–187; UniProt ID: Q9UNE0) which interacts with EDA-A and its potential Death Domain (DD) in the intracellular cytoplasmic region (residues from 209–448; UniProt ID: Q9UNE0), which is considered to function by associating with EDARADD, an adaptor protein of EDAR via its DD (residues from 123–202; UniProt ID: Q8WWZ3), leading to the downstream activation of NF-κB [18,52]. The novel missense variant (p.Trp434Gly) was reported in the DD of EDAR, which interacts with DD of EDARADD (BioGRID ID: 116118) [52]. Due to the unavailability of the crystal structure, EDARADD (Death domain) was modeled using the same procedure as described above. The docked complexes from the highest cluster were selected for further structural analysis. Protein function and the stability effect upon alteration (ΔΔG) was estimated using the DUET server [52,77], an integrated computational approach that combines two complementary approaches (mCSM and SDM). Furthermore, the thermal stability (ΔΔG), resulting from vibrational entropy changes (ΔΔS), was also estimated using the Elastic Network Contact Model (ENCoM) server [36].

### 4.6. MD Simulations and Binding Free Energy Calculations

MD simulations were performed in two steps: (1) 20ns MD simulation to refine and optimize the homology models (wild-type and mutants) before the protein docking, (2) a second 100ns MD simulations to monitor the all backbone atoms stability of WNT10A/Fz8-CRD and EDAR/EDARADD complexes (wild-type and mutants). 

All simulations were carried out by AMBER 16 [78] using the same protocol as described elsewhere [29]. The complexes were analyzed using Chimera 1.13 [79]. The coordinate trajectories were collected after every 2ps for the complete 100ns production run, and the CPPTRAJ module of AMBER16 was used to analyze the trajectories [80]. Molecular Mechanics-Generalized Born Surface Area (MM-GBSA) were calculated using 1000 snapshots, extracted from the whole trajectory. The MM-GBSA approach is well-documented in the binding free energy calculations [81].

## 5. Conclusions

In this research work, we have performed in-depth genetic analyses by using state of the art techniques for the identification of pathogenic variants in the novel and already known genes to solve the fifty-seven distinct tooth agenesis cases. We have identified six pathogenic variants in *WNT10A*, *EDAR*, and *EDA* in eighteen patients of six genetic pedigrees (A-F), exhibiting syndromic and non-syndromic missing teeth phenotypes. The proteins encoded by these genes regulate the *NF-kB* and *Wnt/β*-catenin signaling pathways, which govern the morphogenesis of human ectodermal appendages. This study verifies the fact, collected by other authors around the world, that pathogenic variants in *WNT10A*, *EDAR*, and *EDA* are the most frequently known causes of autosomal recessive hypodontia/oligodontia, autosomal recessive HED, X-linked HED, and X-linked isolated tooth agenesis.

## Figures and Tables

**Table 1:** Structure validation of homology models using Molprobity and ProSA z-score.

Models Using SWISS-MODEL	Molprobity				ProSA z-Score
	Molprobity Score	Ram.Fav (%)	Ram.Out (%)	Rot.Out (%)	
**WNT10A-wt**	1.45	91.3	2.81	2	−6.82
EDAR-wt	1.98	92.94	2.35	2.6	−6.34
EDARADD	1.82	90.91	3.21	2.84	−6.02

Ram.Fav: Ramachandran Favored; Ram.Out: Ramachandran Outliers; Rot.Out: Rotamer Outliers. Molprobity score represents a single score as calculated from clash-score, Ramachandran, and Rotamer evaluations; wt: wild-type.

**Table 2:** Estimated protein structure quantitative stability changes upon alterations.

Mutated Models	DUET	ENCoM	
	Consensus Prediction from mCSM and SDM (ΔΔG kcal.mol^−1^)	Vibrational Entropy Energy (ΔΔS vib kcal.mol^−1^.K^−1^)	Thermal Stability (ΔΔG kcal.mol^−1^)
WNT10A-mt (Arg104His)	−1.431 (destabilizing)	0.523 (increase in flexibility)	−0.348 (destabilizing)
EDAR-mt (Trp434Gly)	−2.762 (destabilizing)	1.661 (increase in flexibility)	−1.329 (destabilizing)

## Supplementary Materials

Supplementary materials can be found at https://www.mdpi.com/1422-0067/20/21/5282/s1.
