# Supplementary material for: Deleterious Variants in WNT10A, EDAR, and EDA Causing Isolated and Syndromic Tooth Agenesis: A Structural Perspective from Molecular Dynamics Simulations
Source: Int J Mol Sci. 2019 Oct 24;20(21):5282. doi: 10.3390/ijms20215282 (PMC6862269; doi:10.3390/ijms20215282)
Supplement: Supplementary file 1 [file ijms-20-05282-s001.pdf]

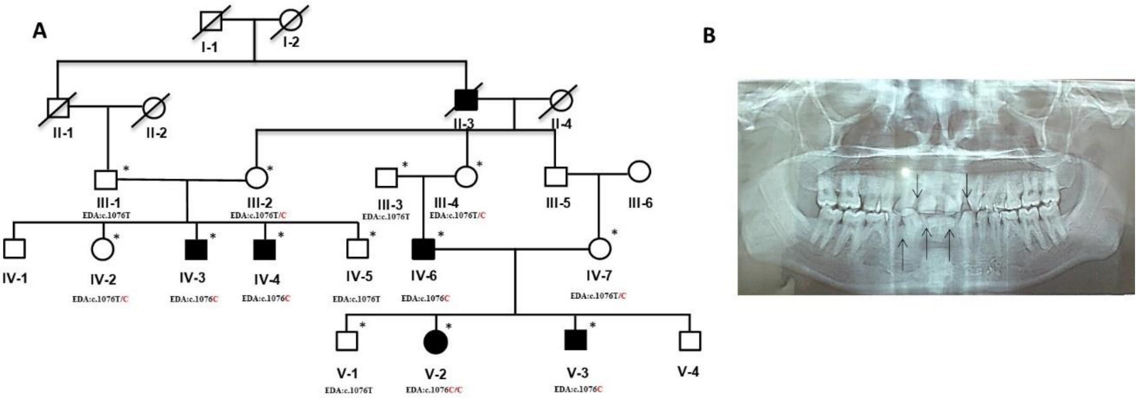

Figure S1: Pedigree of Family D showing X-linked mode of inheritance. **(A)** Asterisks (*) are representing the DNA samples available for study. **(B)** Orthopantomogram (OPG) analysis of the affected member (IV-4) is showing the absence of lateral incisors in the maxilla and mandibular, central incisors of mandibular, second premolars (teeth # 12, 22, 32, 42, 31, 41, 35, 45 respectively). The presence of some retained deciduous teeth is also visible (teeth # 65, 71, 73, 75, 81, 85). The segregation of disease-allele (red) and wild-type allele (black) is shown below the each participant.

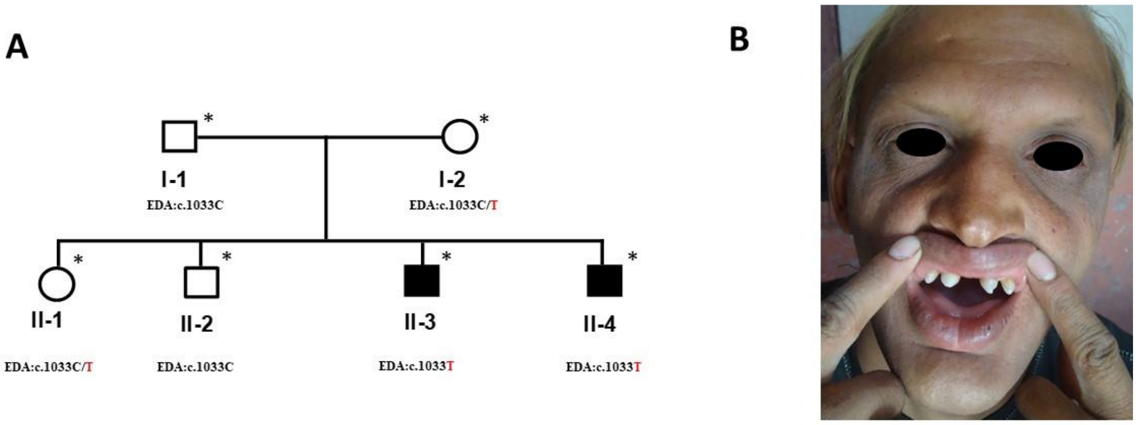

Figure S2: Pedigree diagram of Family E showing X-linked HED. (A) Asterisks (*) are representing the DNA samples of available affected and unaffected members. (B) Patients (II-3) is showing typical canonical teeth, saddle-shaped nose, and pigmentation around the eyes. The segregation of disease-allele (red) and wild-type allele (black) is shown below the each participant.

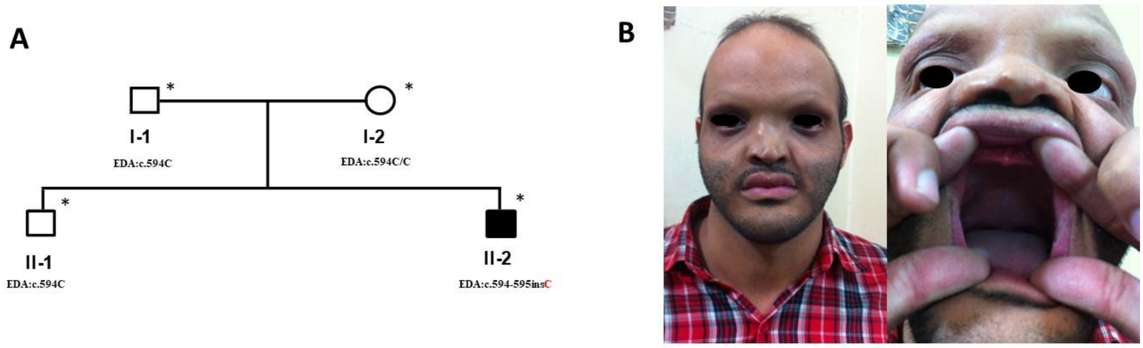

Figure S3: Pedigree of X-linked HED family F. (A) Available samples are labeled with asterisks (*). (B) Affected member (II-2) is showing sparse eye-brows and eye-lashes, prominent lips, and absent frontal teeth. The segregation of disease-allele (red) and wild-type allele (black) is shown below the each participant.

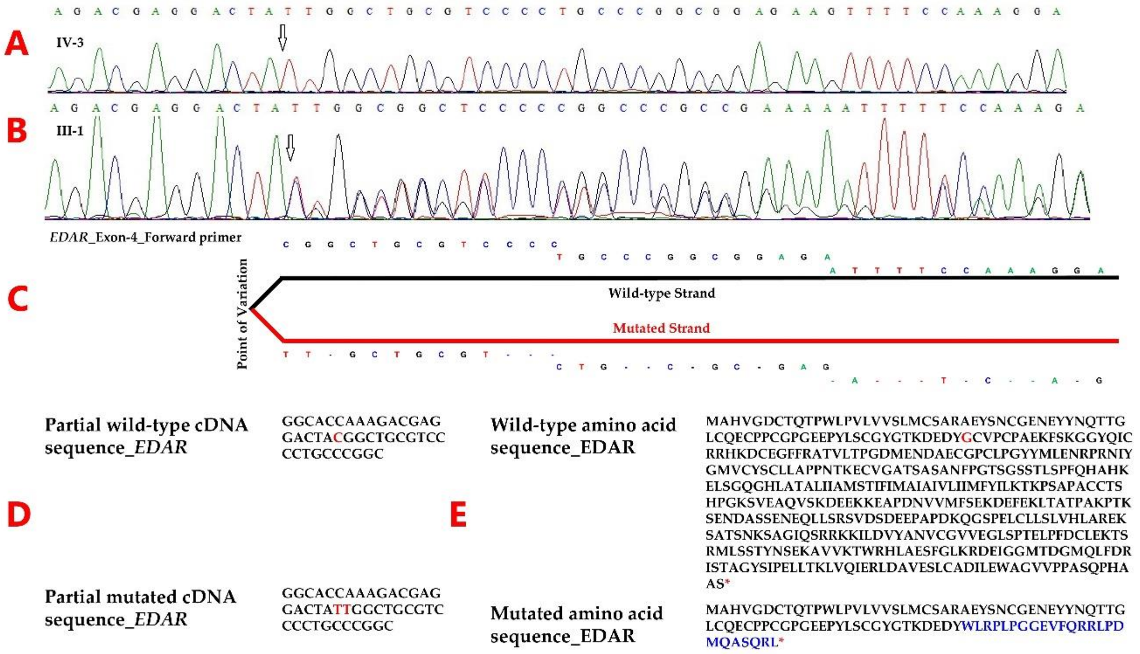

Figure S4: **Panel A:** B are showing the nucleotide sequences of an affected (IV-3) and a carrier individual (III-1) of family B in the result of a frameshift variant c.207delinsTT, p.Gly70Trpfs*25 in EDAR (a similar sequence can be found in Figure 4B in the main text). Panel C is showing wild-type and mutant DNA strands after the point of complex genetic alteration (homozygous deletion of C nucleotide and homozygous insertion of TT nucleotides at the same point). Panel D and E are the cDNA and protein sequences of EDAR, obtained from MutationTaster. Panel D is showing wild-type cDNA sequence and mutated cDNA sequence, red C is the deleted nucleotide, and red TT are the two inserted bases. Panel E is presenting the wild-type and mutated amino acid residues. Red G (Gly) is the point of alteration, and the blue amino acid sequence shows the protein composition after the variation leading to a premature termination codon, thus forming a truncated protein (Predictions from MutationTaster, http://mutationtaster.org/).

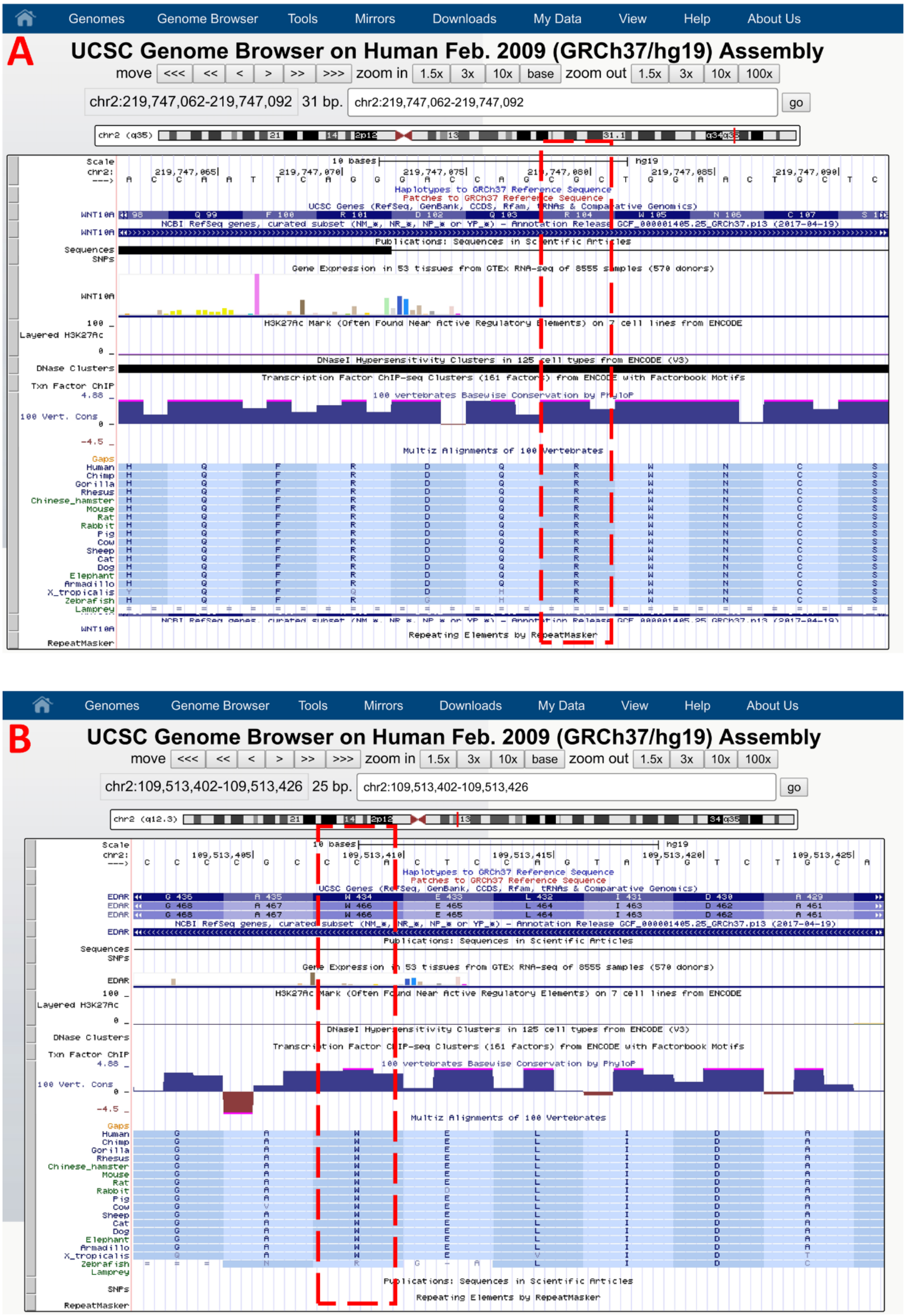

Figure S5: Evolutionary conservation of amino acids Arg104 (A) of WNT10A (family A) and Trp434 (B) of EDAR (family C). The images are taken from UCSC Genome Browser.

Table S1. Protein-Protein Docked complexes of *WNT10A/Fz8-CRD* and *EDAR/EDARADD.*

WNT10A/Fz8-CRD				***EDAR/EDARADD* docked complex**			
**Cluster**	**Members**	**Representative**	**Weighted Score**	**Cluster**	**Members**	**Representative**	**Weighted Score**
**0**	90	Center	-1609.9	**0**	137	Center	-737.6
		Lowest Energy	-1632.9			Lowest Energy	-682.4
**1**	80	Center	-1569.4	**1**	98	Center	-658.7
		Lowest Energy	-1668.8			Lowest Energy	-639.4
**2**	53	Center	-1491.1	**2**	64	Center	-571.1
		Lowest Energy	-1544.6			Lowest Energy	-634.5
**3**	51	Center	-1436	**3**	59	Center	-559.7
		Lowest Energy	-1579.1			Lowest Energy	-537.8
**4**	50	Center	-1421.7	**4**	55	Center	-575.2
		Lowest Energy	-1666.5			Lowest Energy	-667.5
**5**	48	Center	-1549.9	**5**	52	Center	-557.8
		Lowest Energy	-1598.4			Lowest Energy	-616.1
**6**	44	Center	-1443.1	**6**	50	Center	-589.1
		Lowest Energy	-1604.2			Lowest Energy	-666.8
**7**	35	Center	-1484.3	**7**	48	Center	-624
		Lowest Energy	-1574.5			Lowest Energy	-681.2
**8**	35	Center	-1407	**8**	46	Center	-602.2
		Lowest Energy	-1509.3			Lowest Energy	-662
**9**	32	Center	-1432.7	**9**	38	Center	-592.6
		Lowest Energy	-1644.8			Lowest Energy	-609
**10**	32	Center	-1558	**10**	36	Center	-548.9
		Lowest Energy	-1680.5			Lowest Energy	-708.3
**11**	25	Center	-1447.4	**11**	36	Center	-557.5
		Lowest Energy	-1521.5			Lowest Energy	-604.1
**12**	22	Center	-1411.6	**12**	36	Center	-548.8
		Lowest Energy	-1504.7			Lowest Energy	-592
**13**	21	Center	-1416.7	**13**	26	Center	-552.9
		Lowest Energy	-1497.8			Lowest Energy	-598.5
**14**	19	Center	-1554.2	**14**	23	Center	-589.9
		Lowest Energy	-1614.4			Lowest Energy	-628.3
**15**	18	Center	-1426.1	**15**	20	Center	-596.9
		Lowest Energy	-1440.2			Lowest Energy	-596.9
**16**	17	Center	-1403.1	**16**	18	Center	-606.8
		Lowest Energy	-1574.8			Lowest Energy	-606.8
**17**	16	Center	-1396.6	**17**	17	Center	-567.5
		Lowest Energy	-1508.1			Lowest Energy	-625
**18**	16	Center	-1479.6	**18**	17	Center	-560.6
		Lowest Energy	-1490.2			Lowest Energy	-579.8
**19**	16	Center	-1563.8	**19**	16	Center	-589.7
		Lowest Energy	-1563.8			Lowest Energy	-589.7
**20**	15	Center	-1395.2	**20**	16	Center	-590.4
		Lowest Energy	-1500.7			Lowest Energy	-590.4
**21**	15	Center	-1416.4	**21**	15	Center	-642.2
		Lowest Energy	-1527.5			Lowest Energy	-642.2
**22**	14	Center	-1398.8	**22**	14	Center	-571.7
		Lowest Energy	-1511			Lowest Energy	-571.7
**23**	14	Center	-1411.1	**23**	13	Center	-574.2
		Lowest Energy	-1498			Lowest Energy	-574.2
**24**	13	Center	-1394.3	**24**	10	Center	-576.9
		Lowest Energy	-1466.8			Lowest Energy	-594
**25**	13	Center	-1425.8	**25**	10	Center	-573.7
		Lowest Energy	-1441.3			Lowest Energy	-607
**26**	13	Center	-1473.6	**26**	9	Center	-547.9
		Lowest Energy	-1474.2			Lowest Energy	-636
**27**	12	Center	-1401.8	**27**	5	Center	-571.6
		Lowest Energy	-1451.4			Lowest Energy	-573.2
**28**	12	Center	-1425.7	**28**	4	Center	-553.5
		Lowest Energy	-1497.2			Lowest Energy	-565.3

**29**	12	Center	-1406.2	**29**	2	Center	-550.7
		Lowest Energy	-1489.2			Lowest Energy	-561.3

Table S2. Primers for variants in *WNT10A* and *EDA.*

	Variant	Primer	Temperature	Sequence
1	WNT10A_c.311G>A_p.Arg104His	F	61.8 C	ggatgtgcataggaaatactgtgg
		R	62.6 C	aggcagtgggttagaaatggc
2	EDA_c.1076T>C_p.Met359Thr	F	63.2 C	gtggtggatgagaagcccttc
		R	65.3 C	tgcggctgcaacaccaatac
